# Nociceptive pain and anxiety in equines: Physiological and behavioral alterations

**DOI:** 10.14202/vetworld.2021.2984-2995

**Published:** 2021-11-26

**Authors:** I. Hernández-Avalos, D. Mota-Rojas, J. E. Mendoza-Flores, A. Casas-Alvarado, K. Flores-Padilla, A. E. Miranda-Cortes, F. Torres-Bernal, J. Gómez-Prado, P. Mora-Medina

**Affiliations:** 1 Department of Biological Sciences, Clinical Pharmacology and Veterinary Anesthesia, Faculty of Higher Studies Cuautitlán FESC, Universidad Nacional Autónoma de México, State of Mexico 54714, Mexico; 2 Neurophysiology of Pain, Behavior and Assessment of Welfare in Domestic Animals, DPAA, Universidad Autónoma Metropolitana, Mexico City 04960, Mexico; 3 Equine Hospital Faculty of Higher Studies Cuautitlán FESC, Universidad Nacional Autónoma de México, State of Mexico 54714, Mexico; 4 Department of Livestock Sciences, Animal Welfare, FESC, Universidad Nacional Autónoma de México, State of Mexico 54714, Mexico

**Keywords:** anxiety, equines, nociception, pain, welfare

## Abstract

Pain and anxiety are two of the most important concerns in clinical veterinary medicine because they arise as consequences of multiple factors that can severely affect animal welfare. The aim of the present review was to provide a description and interpretation of the physiological and behavioral alterations associated with pain and anxiety in equines. To this end, we conducted an extensive review of diverse sources on the topic. The article begins by describing the neurophysiological pathway of pain, followed by a discussion of the importance of the limbic system in responses to pain and anxiety, since prolonged exposure to situations that cause stress and pain generates such physiological changes as tachycardia, tachypnea, hypertension, hyperthermia, and heart rate variability (HRV), often accompanied by altered emotional states, deficient rest, and even aggressiveness. In the long term, animals may show deficiencies in their ability to deal with changes in the environment due to alterations in the functioning of their immune, nervous, and endocrinologic systems. In conclusion, pain and anxiety directly impact the homeostasis of organisms, so it is necessary to conduct objective evaluations of both sensations using behavioral scales, like the horse grimace scale, complemented by assessments of blood biomarkers to analyze their correlation with physiological parameters: Heart rate, respiratory rate, HRV, theparasympathetic tone activity index, lactate and glucose levels, and temperature. Additional tools – infrared thermography, for example – can also be used in these efforts to improve the quality of life and welfare of horses.

## Introduction

Pain is one of the most important concerns in the clinical veterinary medicine of equines because it is a consequence of multiple factors that impede achieving the goals established at breeding units and directly impact the welfare of animal species. Equines present an especially difficult challenge because this species has evolved toward stoic behaviors, presumably in efforts to evade predation. These include using alarm signals to alert other animals to the presence of a dangerous individual and allowing them to take measures to deal with the threat [1-3]. In this regard, it is important to mention that in horses, during the clinical recognition of pain, various factors intervene, including the exposure time, type, and location of the pain, since in prolonged exposures the animal will develop behavioral changes that will be influenced by age, sex, temperament, and even work done [4,5]. This sign is a physiological process that, Reid *et al*. [6] have identified as two kinds of pain in relation to the time of evolution. Acute pain is the initial response to the activation of peripheral nociceptors due to thermal or physical stimulation that is of short duration and is characterized by being self-limiting. Whereas, chronic pain refers to that which persists for 3-6 months or longer, which evolves gradually [7]. But is capable of reducing the quality of life and animal welfare [8,9].

The second sensation analyzed, anxiety, refers to an emotional response characterized by uneasiness and fear, accompanied by an autonomous response caused by the anticipation of danger [10]. In this case, the potential threat is not immediately present, but results from continuous exposure to some stressful factor (stressor) [6,11]. As a species, horses are highly susceptible to suffering problems related to anxiety. Harro [12] states that these disorders are elements of a problem that significantly affects young and adult animals, predisposing them to develop neurological and cardiovascular diseases, and accelerated aging, while also reducing their quality of life and level of welfare. Stuijfzand *et al*. [13] mentioned that animals with high anxiety levels show hyperreactivity to threatening, moderate, or even ambiguous stimuli, with attentional biases that facilitate the detection of threats and taking aversive action. For this reason, these animals are more prone to manifesting behavioral or physiological alterations induced by stress [14].

In addition, prolonged exposure to stressful or painful situations decrease the level of well-being enjoyed by the equine, causing physiological and behavioral changes that will be reflected in the increased of physiological parameters, negative emotional states, and show deficiencies to cope with changes in the environment that surrounds it due to a reduced functioning of their immune, nervous, endocrinologic, and limbic systems [9,15]. These changes can be evaluated by measuring chemical substances secreted in the blood, such as adrenaline, noradrenaline, and cortisol, among others, to prepare individual animals to confront situations that endanger their integrity [16,17]. In this way, pain and anxiety are considered negative emotions [18,19]. In equines, both experiences can be caused not only by a clinical pathology, but also by tissue damage due to a traumatic lesion, infectious disease, neoplasia, or during an inflammatory response [20]. It is in this context that the present review describes and interprets the physiological alterations associated with pain and anxiety in equines, including heart rate, respiratory rate, heart rate variability(HRV), temperature, and endocrine disorders, as well as the behavioral patterns that these animals manifest under these conditions.

## Neurophysiological Pathway of Pain

**Figure-1:**
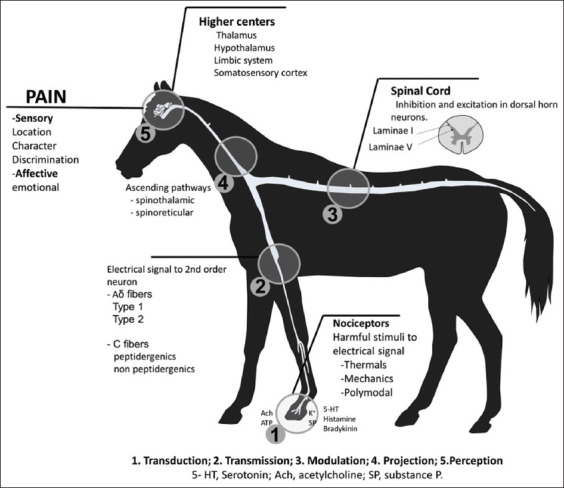
Neurophysiological pathway of nociceptive pain [8,27-29].

The International Association for the Study of Pain defines this phenomenon as an unpleasant sensory and emotional experience associated with potential tissue damage, or an experience described in terms of such damage [21-23]. As in other species, in equines, this aversive sensory and emotional experience reflects the fact that the animal is conscious of potential damage or a threat to the integrity of its tissues that causes it to produce physiological and behavioral changes as it attempts to reduce or eliminate the damage, prevent its reappearance, and promote recovery. Unnecessary pain occurs when these physiological or behavioral responses are unsuccessful [24]. Pain is, therefore, a complex disorder that involves sensory, motor, emotional-affective, and cognitive components [25,26] that can be represented visually in the nociceptive arch (Figure-1) [8,27-29].

The neurophysiological pathway of pain originates in the nociceptors that, under physiological conditions, reach their activation threshold only in the presence of potentially harmful stimuli that are capable of causing tissue damage [27,30,31]. Nociceptors are stimulated by substances released in injured tissues, such as potassium ions, bradykinin, histamine, substance P, serotonin, acetylcholine, and ATP. Studies have demonstrated that various members of the family of transient potential and acid-sensing ionic channels are also important in generating action potentials derived from harmful stimuli [32-35]. This first step in the process is called transduction [26]. In situations of hypersensitization, such as allodynia or hyperalgesia, however, nociceptors can be activated even by innocuous stimuli [36-39].

Hector and Mama [8] mention the existence of three types of nociceptors: thermal, which are activated at temperatures >45°C or <5°C and conduct impulses through type Aδ myelinated fibers; mechanical, which respond to tactile stimuli and also send impulses through type Aδ myelinated fibers; and polymodal, which respond to various mechanical, thermal, and chemical stimuli.

The second step in the process is called nociceptive transmission. This takes place through Aδ fibers that are related to the sensation of localized, acute pain, and is accompanied by the avoidance reflex [40,41]. Electrophysiological studies classify these neurons in two main types: Type 1, HTM: High threshold mechanical nociceptors, that respond to stimuli with an elevated threshold (e.g., 50°C); and type 2, which have a lower heat threshold, but maintain an elevated threshold towards mechanical stimuli [42,43]. The information carried by the latter moves more slowly because it is transmitted through the activity of C fibers, which give a persistent, but poorly located, sensation that is called burning pain [27]. These type C fibers have been classified as peptidergic and non-peptidergic. The former is related to substance P, a peptide associated with the gene of calcitonin gene-related peptide and somatostatin [44,45].

The transmission of the nervous impulse is effectuated when the bodies of peripheral axons are in the ganglia of the dorsal roots and their central prolongations form synapses with second-order neurons in the dorsal horn of the spinal cord, where painful stimuli from Aδ and C fibers are recognized [8]. A previous study demonstrated that laminae I and V are the best exit points for projections towards higher centers [46], as they lead to such structures as the hypothalamus, thalamus, limbic system, and certain cortical areas, primarily SI and SII. These structures codify the information to produce the real, conscious experience of pain (Figure-1) – whether sensory (localization, character, discrimination) or affective (emotional) – which occurs when the nervous impulse reaches the specific brain structures (hypothalamus and thalamus) that modulate nociception [29,47-49].

From a neurophysiological perspective, pain is divided into nociceptive and non-nociceptive forms. The first results from the activation or sensibilization of nociceptors in the periphery that transduce the harmful stimulus, and concludes with activation of various supraspinal objectives that modulate the nociception and lead the individual to experience pain. The latter can result from a lesion in the neuronal structures of the peripheral and central nervous system (CNS), where it triggers aberrant somatosensorial processing [21,27,50].

## Importance of the Limbic System in Response to Pain and Anxiety

**Figure-2:**
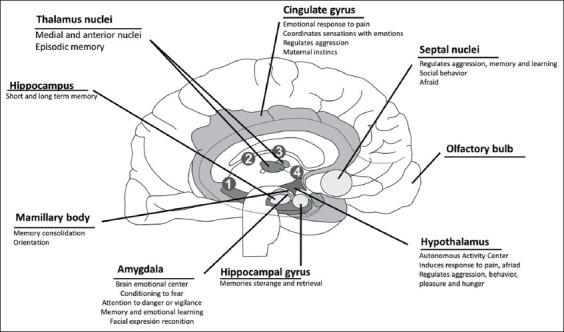
The limbic system and its participation in sensations of pain and anxiety [51].

System limbic plays an essential role because it receives sensory information such as visual, auditory, tactile, and olfactory stimuli, which causes the activation of intercommunication pathways within this system, represented in Figure-2 [51], where it has been demonstrated neuroimaging cortex, insula, cingulate cortex, thalamus, and periaqueductal substance are activated [52]. In the same way, the amygdala can be highlighted, which forms connections with the olfactory bulb, hypothalamus, and somatosensory cortex that transmit peripheral signals to carry out emotional and physiological processing during stress responses, which includes pain or anxiety. López Mejía *et al*. [53] describe, for humans and animals, how it was possible to establish that the amygdala intercommunicates with the system for the somatic expression of emotions (hypothalamus and nuclei of the brainstem). They consider this the system of concise sentiments, especially fear, which involves the cingulated, parahippocampal, and frontal cortices. Various conditions – anxiety, depression, post-traumatic stress syndrome, and phobias, among others – are linked to abnormal functioning of the amygdala due to injury, developmental disorders, or imbalances of various neurotransmitters [54].

The amygdala forms part of the limbic system. It is located in the medial temporal lobe, which is known for its role in the emotional states of sensory stimuli, related to behavioral adaptations in response to changes in an organism’s internal and external environment [49,55,56]. Current lines of research, both anatomical and physiological, suggest that nociceptive projections that originate in the lamina I of the dorsal horn of the spinal cord, or the spinal nucleus of the trigeminal nerve, leading to the parabrachial nucleus and then to the central nucleus of the cerebral amygdala, which functions as the main exit for projections from the amygdala, performs functions related to pain, and participates significantly in the emotional-nociceptive component [57] by modulating part of the behavioral component, including, to a large degree, facial expressions generated by pain [49,58-61]. Schmidt *et al*. [62] evaluated the morphology of the warm blood horse through magnetic – resonance - imaging; they found functional connections located mainly in the limbic system between cortex somatosensory and amygdala, areas that have been proven to be responsible for threat detection [63].

The CNS has neuronal groups called central pattern generators (CPG), located in the mesencephala, bridge, and spinal cord, in both humans and animals. These groups form part of the neuronal circuits that organisms possess (i) to modulate their adaptation to the demands of the environment, and (ii) allow individuals to express motor responses that include recognition of emotions [64]. CPG are activated principally by stimulation of the peripheral sensory receptors and signals generated by other nuclei of the CNS. As a result, the limbic system regulates the expression of emotional responses, while the CPG associated with this system initiate and controls the activity of the facial muscles to generate a conservative, stereotypical response to a specific stimulus [65,66].

During the sensations of fear and terror, the peripheral stimuli will lead to the activation of the limbic system that will produce physiological responses such as increased heart rate and respiratory rate, dry mouth, muscular tension, and sweating [67,68]. In the same way, there is an emotional reaction that communicates the internal state of an individual that can be reflected through gestures or facial expressions [19]. Previously, it was mentioned that emotions were uniquely human characteristics; however, Charles Darwin described them as emotional states such as fear, aggression, and pain in animals expressed by postural reactions and facial expressions [53,69]. Tortora and Derrickson [51] demonstrated that when certain areas of an animal’s limbic system are stimulated (Figure-2), subject’s reactions can run from intense pain to extreme pleasure. They further described that stimulating other areas of the limbic system causes docility and signs of affection. This system, in conjunction with frontal cortex structures, processes emotional stimuli, and integrates them into more complex cerebral functions [53,70,71].

Understanding an emotion such as fear requires, first, knowing the relationship between the cognitive sentiment represented in the cerebral cortex and the associated physiological signs regulated by subcortical areas [46,72]. This process occurs in the following order. First, an emotional stimulus of significant intensity activates sensory systems that send information to the hypothalamus. This area generates a response that is capable of modulating heart rate, blood pressure, and respiratory rate. At the same time, the information from this stimulus is carried to the cerebral cortex; hence, it is taken indirectly from the peripheral organs (which lose their homeostatic state due to the stimulus) and directly from the hypothalamus, amygdala, and related structures [73]. Hence, from a neurobiological point of view, anxiety, and pain share the communication pathways in the limbic system that makes it difficult to establish a difference for their recognition since they will lead to similar physiological reactions. Therefore, the limbic system fulfills the role of interconnection, processing, and response, both physiological and emotional, which will be reflected with the activation of the sympathetic nervous system or postural reactions that communicate the internal state of the individual.

## Physiological Alterations in Heart Rate and Respiratory Rate associated with Pain and Anxiety

When subjected to processes involving pain or anxiety, equines initiate a normal physiological response to these stressful stimuli. This response can be evaluated by measuring alterations in the vital signs, such as heart rate and respiratory rate [74]. König *et al*. [20] mention that when a situation is perceived as a stressful factor, signals are sent through the efferent nerves to mediate the physiological responses of the somatic and autonomous nervous systems. This results in behavioral responses such as fight or flight.

Physiological responses entail activating two principal pathways: The hypothalamic-pituitary-adrenal cortex axis (HPA) and the ­sympathetic-adrenomedullary (SAM) axis. The latter generates immediate responses through activation of the sympathetic branch of the autonomous nervous system, causing the systemic release of catecholamines (epinephrine and norepinephrine) that stimulate glycogenolysis. Epinephrine produces an increased heart rate and dilatation of the arteries to improve blood flow in the vital organs, while norepinephrine causes the constriction of veins and arteries in non-vital organs, leading to tachycardia, hypertension, and reduced gastrointestinal activity [20,74-77].

This response is not, however, exclusive to nociceptive pain, because anxiety can also activate the HPA axis and produce changes such as fluctuations in adrenalin and cortisol levels that affect heart rate [6,78]. In this regard, and in reference to horses, Von Borell *et al*. [79] mention that pain and anxiety not only alter heart rate but also increase respiratory cycles, blood pressure, and the interval between heartbeats. Some results in horses are, however, controversial and suggest that these parameters alone are not effective in determining, objectively, the presence or severity of pain [80-84].

## Endocrine Alterations Associated with Pain and Anxiety Improve

Exposing equines to stressors provokes negative emotional changes [20] that trigger a physiological response as the animals seek to recover homeostasis [74,85-87]. These endocrine changes are produced by the activity of the SAM and HPA systems [18,25,88,89], which are responsible for regulating such biological functions as immune competency, reproduction, metabolism, and behavioral aspects, among others [90,91].

The activation of the SAM system, by itself, causes an increase in cardiovascular parameters due to the neurosecretion of catecholamines by activating the adrenoreceptors located in the vascular endothelium [92,93]. This will generate an increase in blood pressure and tachycardia during acute pain perception[93]. In this sense, Gehlen *et al*. [94] evaluated the cardiovascular and endocrine response of 43 horses with colic over time (admission, 24 h and discharge) against three forms of management (surgical, conservative, and sacrifice). It was observed that HRV and cortisol levels were greater, as well as the cortisol levels, during the admission of the animals but this response decreased after 24 h of the onset of the event, which according to the authors is due to a decrease in the sympathetic response, although there is a primary response of the autonomic nervous system that helps to respond by preparing the organism to an alert event, these indicate that said response cannot be sustained but could serve to issue a survival prognosis in animals under severe conditions as shown. It was observed in 51 equines that under 1 year of age diagnosed with colic, where a HRV was greater differences between RR intervals in animals that had a greater probability of surviving compared to animals that had a lower probability of survival (64.8 ms vs. 33.4 ms) [95]. The previous observations confirm that this primary response to stress or pain is unlikely to be a sustained response that could be the prelude to late endocrine changes [96,97].

This suggests that some endocrine changes can be utilized as indicators of stress, as in the case of measuring cortisol concentrations in plasma, saliva, urine, feces, or hair [2,18,20,98]. However, Becker-Birck *et al*. [99] observe that the release of these indicators will vary with the type of stressor and time of exposure, such that cortisol levels in plasma and saliva reflect acute changes, while in hair and feces they indicate situations of prolonged stress. The latter approaches have the advantage that sampling is non-invasive [100-102].

Because the HPA axis is associated with long-term effects, it is involved in secreting the corticotropin-releasing hormone (CRH) in the hypothalamus, where corticotropes are stimulated by CRH to trigger the release of the adrenocorticotropic hormone (ACTH) in the pituitary gland, or hypophysis which, in turn, stimulates the secretion of corticosteroids such as cortisol and corticosterone from the adrenal cortex [20,103,104]. These steroids then trigger catabolic processes of glycolysis, lipolysis, and gluconeogenesis [105,106].

Cortisol and its metabolites are the analytes most often utilized to evaluate the response of the HPA axis [18]. During short-term stress, glucocorticoids improve energy mobilization by increasing the release of cortisol which, since it does not bond to proteins, diffuses rapidly from the blood to the saliva [74,107], so it can be used as an indicator of stress. Hall *et al*. [87] evaluated saliva cortisol levels by immunosorbent assay in ten equines during training together with an evaluation of behavioral elements and ocular temperature. They found variations among individuals due to a correlation between concentration and the age of the horse, as well as the differing physical demands of each animal’s activity. Their results coincide with findings from various other authors which suggest that situations that provoke anxiety also produce increases in saliva cortisol concentrations [87,107,108]. In addition, according to a recent study, it has been observed that the experimental stimulation of this axis with ACTH is associated with the increase of cortisol, aldosterone, androstenedione, pregnenolone, 17a-OH-progesterone, and progesterone in critically ill patients compared to healthy animals. But interestingly, it was observed that in the animals that died, a decrease in cortisol and an endogenous increase in ACTH were observed, which suggested adrenocortical dysfunction [109]. This corroborates what has been stated above, first that the stimulation of cortisol decreases the response of the immune system, while second, this endocrine response has a limited response that can be depleted, which can serve as a prognostic factor in severe conditions.

Hence, the use of these indicators should be taken with caution; in this context, König *et al*. [20] suggest that to ensure that the measurements taken are significant and representative of hormonal responses to stress, samples must be drawn before and after the onset of the stressful stimulus. Other authors, in contrast, have concluded that endocrine measurements can reflect responses to stress that are not pain-related [110,111], while also indicating the duration or severity of pain [112]. Although there may be justification for including these endocrine measurements, they are not reliable parameters as unique indicators of pain in horses [2]. It is argued that there may be a variable in relation to the breed, as was observed in a comparative study between the response of the adrenal glands between ten purebred and standard breed foals where it was observed that the levels of cortisol, progesterone, adrenocorticotropin, and aldosterone were higher in purebred animals compared to standard breed animals, as well as a significant difference in the size of the adrenal medulla between these animals, which suggests a characteristic to consider for the evaluation of this analyte [113].

Other indicators that have been employed with equines include blood glucose [104], and lactate levels [114], serum creatine kinase [115], and plasma beta-endorphin [116,117], but these parameters are not exclusive for detecting stress [20,97]. Therefore, an endocrinal response can be an indicator of anxiety and pain that could even help to identify when it is a chronic or acute event by considering the indicators involved. However, further research is necessary.

## Perspectives on Using the Index of Parasympathetic Tone Activity (PTA) in Equines

Studies of HRV and PTA, and their relation to hemodynamic changes in anesthetized horses, have been carried out by monitoring and measuring the PTA index. Unfortunately, results to date have been discrepant. A study of 39 horses by Mansour *et al*. [118] evaluated the clinical state and performance of this index for predicting variations in blood pressure. They reported that horses with acute abdominal syndrome presented low scores on the PTA index compared to animals admitted for elective procedures. This could reflect a predominant sympathetic tone as a consequence of pain and the animals’ critical condition; though mean blood pressure values between groups showed no statistical differences. The increase observed in the PTA scores did, however, show a correlation with a reduction in blood pressure in the ensuing 5 min. This leads to the conclusion that the PTA index can provide information on the sympathovagal balance and the degree of analgesia/nociception in anesthetized animals, and may have potential use as a prognostic factor in horses in critical condition.

Although the PTA index has been described as a tool that can contribute to analyses of intraoperatory nociception –not only in animals – Ruíz-López [119] determined that PTA values in anesthetized horses admitted for elective processes showed no significant changes after a surgical stimulus, and that their correlation with heart rate and blood pressure media was weak or null in both cases. Hence, the conclusion for this case was that, although the PTA values were not modified after administering dobutamine and morphine, further studies are required to determine the usefulness of this index in clinical contexts for monitoring nociceptive pain, with the goal of providing more compassionate attention to pain through enhanced analgesic protocols.

## Behavioral Patterns and Facial Expressions in Equines Associated with Pain and their Significance

The most frequent causes of pain in equines involve abdominal pathologies, disorders of the extremities, and dental abnormalities [120,121], conditions that may be manifested in postural changes, facial expressions, feeding alterations, and poor interaction with conspecifics that can lead to social isolation and even estrangement from humans, all of which augment the potential for physical damage to farm personnel during handling. This coincides with the position adopted by Zimmerman [122], which holds that pain “provokes protective motor actions that result in learned avoidance and that can modify the specific behavioral traits of the species, including its social behavior.”

That study was carried out using scales in which an evaluator assigned scores based on observation of, and interaction with patients. The aim was to quantify the intensity of pain according to the level of incapacity of movement that the animal manifested, as follows: Sore, limiting, defensive to touch in the area around the lesion, presence of trembling, complete paralysis, and exhaustion [123-125].

A recent, objective assessment of pain set out to study the behavior of animals based on the hypothesis that framing unpleasant experiences and emotions gives rise to changes – subtle or more evident – in posture, march, and behavioral interaction. The behavior of horses, however, can be influenced by multiple factors, including breed, temperament, sex, and familiarity with the environment [126,127]. All these phenomena can affect anxiety levels and pain thresholds in equines [128].

It is also important to consider the existence of specific and unspecific factors of pain in equines, both of which may be reflected in behavioral changes expressed as depression and anxiety [129-131]. Some horses subjected to painful events look away, seem distracted, and have difficulty in finding a comfortable resting position, so they spend more time standing in an alert state. However, these behavioral alterations will differ depending on the intensity of the pain and/or time of exposure, mainly because an animal may become habituated to pain through an elevation of its pain threshold, possibly attributable to an increase of nociceptors or a decrease of the excitability threshold. These aspects are known as supra- and infra-regulation [17].

Under these circumstances, and with the goal of improving clinical evaluations of pain in equines in mind, researchers have developed scales of facial expressions called “grimace” scales that make it possible to objectively evaluate pain in degrees that run from “mild” to “severe,” instead of simply describing its manifestations [131]. This kind of scale has been used in studies with humans and other species, such as rats, mice, rabbits, sheep, and cats [132-135].

Dalla Costa *et al*. [136] described the “horse grimace scale (HGS)” using six facial action units: ear position, orbital adjustment, tension in the area around the eyes, tension in the chewing muscles, tightness of the mouth, pronounced chin, and flaring nostrils. They assigned independent scores to each facial action unit. At first, the HGS was designed to evaluate pain during elective surgery, as in the case of castration. The conclusion was that facial expressions showed significant differences that were reflected in higher scores for pain in groups of animals that were subjected to surgery compared to those that were not. No significant differences were observed between the analgesic treatment schemes applied pre- and post-surgery.

This same scale was used to analyze its applicability in horses that were suffering from acute laminitis [137]. In this case, observations showed that the HGS is a potentially effective instrument for measuring pain associated with this disease, since comparisons with scores on the Obel scale [138] showed equivalent evaluations.

Gleerup *et al*. [131] performed a controlled study that involved videotaping horses to evaluate their facial expressions in the presence and absence of an observer. Pain was induced with two harmful stimuli (a tourniquet on the forelimb and topical application of capsaicin). Those authors reported that both stimuli increased pain scores, observing greater alterations in the facial expressions in the control group (p=0.001) where the animals exhibited asymmetric/low ears, angled eyes, a tense withdrawn gaze, square flaring of the nostrils, and tension in the muzzle and chewing muscles. These signs can be recognized in horses as expressions of acute pain.

**Figure-3:**
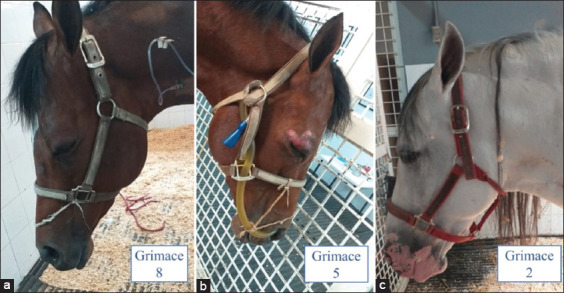
Clinical follow-up of pain based on the horse grimace scale in horses with different degrees of pain. (a) Acute pain severe; it is possible to observe dilation in nostrils, tension in the muzzle and chewing muscles, moreover, an asymmetric position in ears (flattener ears) in a male horse diagnostic with colic. (b) Acute pain moderate; it shows a horse male with colic with treatment for 6 h, where it is possible to see facial movements such as asymmetric/low and accompanied by angled eyes, a tense withdrawn gaze and note the clinical evolution in the position of the ears in comparison with a. (c) 24 h post-treatment. At first, they were, square flaring of the nostrils and tension in the muzzle and chewing muscles. At 24 h post-treatment, however, these scores had decreased. This evidence shows the utility of facial expressions for evaluating analgesic responses.

Recently, Costa *et al*. [139] demonstrated that HGS scores were not modified by emotional states distinct from pain, such as a new environment, grooming, or reward anticipation using food and fear, though in the latter case the scores for rigid, backward-pointing ears and prominent tension in the chewing muscles did register average score of 3. These results show that the HGS is a specific tool for measuring pain in horses (Figure-3).

In addition to the HGS, objective measurements of pain have been conducted using other types of scales based on parameters of facial features. Together, these are known as the “Score Sheet of the Equine Utrecht University Scale for Facial Assessment of Pain” [83]. When evaluating severe states of pain caused by acute abdominal syndrome, this “score sheet” showed high inter-observer reliability (intraclass correlation coefficient [ICC]=0.93, p<0.001) compared to an analogous visual scale (ICC=0.63, p<0.001). Indeed, this instrument was able to differentiate the treatment – conservative versus surgical – in 6 out of 11 horses, achieving a sensitivity of 87.5% and a specificity of 88%.

Another feature closely related to acute pain involves manifestations of aggressiveness. This has been studied mainly in contexts of painful practices that reduce the level of animal welfare because they tend to provoke fear in animals before handling. In this case, fear is manifested in advance of the stimulus because animals associate it with pain and depression in cases of chronic pain [140,141]. Other observations in horses exposed to acute pain reveal that they tend to tighten their jaws, resist handling, and increase the frequency of vocalizations and the Flehmen reflex, with frequent episodes of continuous swaying. On many occasions, this is seen to be associated with more severe levels of pain that, under certain circumstances, may be confused with events of aberrant behavior [1].

When intestinal lesions occur in horses, especially obstructions and strangulations of the digestive tract, it is well known that they tend to roll over on their back and try to kick the abdominal area when pain is acute. In the specific case of donkeys and foals, they turn their heads to look toward their flanks and manifest self-isolation behaviors by tending to stay away from their handlers [142-145]. This behavior reveals the participation of the amygdala and its connectivity with other limbic structures during perceived pain and anxiety, findings similar to those described in rodents that show how stress causes structural and functional remodeling of amygdala neuronal outputs to defined cortical and subcortical target regions [146].

In most cases, it is difficult to correctly identify the pathological signs that these animals manifest, which leads to confusion in reaching diagnoses and delays in treatment onset, as well as iatrogenesis. This underscores the importance of understanding the etiology of both the equine species and the ­pathology involved to reach accurate, differential diagnoses [128]. Therefore, it is necessary to support diagnoses with complementary examinations, such as studies of blood biomarkers and physical and physiological evaluations that include vital variables such as heart rate, respiratory rate, HRV, the PTA index, lactate and glucose levels, and temperature, while also incorporating other mechanisms, such as infrared thermography.

## Conclusion

Increased respiratory rate, hyperthermia, tachycardia, and an increase in HRV are clear alterations in physiological parameters during the perceptions of stimuli that cause stress such as pain or anxiety, and these sensations produce a state of stress in the animals that directly influence homeostasis in the organism and alters that indicate their state of health. Nevertheless, only use of these parameters to identify anxiety or painful states, it is not clear can be differentiated during event states anxiety or pain.

Increased levels of cortisol, lactate, and catecholamines can be considered as endocrine biomarkers during both events, although if they can help to differentiate between an acute event from an event chronic, this does not help to clearly differentiate states of pain or anxiety. Therefore, the physiological parameters and the PTA index need to be evaluated together with endocrine biomarkers, behavioral patterns, and facial expressions that guarantee precise interpretation to improve their quality of life and animal welfare.

## Authors’ Contributions

DM and IH: Conceptualized, drafted, and supervised the final version. DMR, IH, AC, and PM: Contributed to the original draft, data curation, investigation, writing, review, and editing of the manuscript. JEM, KF, FT, JG, and AEM: Worked on the methodology, writing, and editing of the manuscript. JEM, AC, and AEM: Collected relevant literature, wrote, reviewed, and edited the manuscript. All authors have read and approved the final manuscript.
